# Impacts of Strain Variation on Response to Heat Stress and Boldo Extract Supplementation to Broiler Chickens

**DOI:** 10.3390/ani10010024

**Published:** 2019-12-20

**Authors:** Mahmoud M. Abo Ghanima, May Bin-Jumah, Abdel-Moneim E. Abdel-Moneim, Asmaa F. Khafaga, Mohamed E. Abd El-Hack, Ahmed A. Allam, Nagwa I. El-Kasrawy

**Affiliations:** 1 Animal Husbandry and Animal Wealth Development Department, Faculty of Veterinary Medicine, Damanhour University, Damanhour 22511, Egypt; mmyvet2@yahoo.com (M.M.A.G.);; 2 Biology Department, College of Science, Princess Nourah bint Abdulrahman University, Riyadh 11671, BO. Box 24428, Saudi Arabia; may_binjumah@outlook.com; 3 Biological Application Department, Nuclear Research Center, Atomic Energy Authority, Abu-Zaabal 13759, Egypt; aeabdelmoneim@gmail.com; 4 Department of Pathology, Faculty of Veterinary Medicine, Alexandria University, Edfina 22758, Egypt; Asmaa.Khafaga@alexu.edu.eg; 5 Department of Poultry, Faculty of Agriculture, Zagazig University, Zagazig 44511, Egypt; 6 Department of Zoology, Faculty of Science, Beni-suef University, Beni-suef, 65211 Egypt; allam1081981@yahoo.com

**Keywords:** boldo extract, heat stress, growth, blood indices, antioxidant, economic efficiency, broiler strain

## Abstract

**Simple Summary:**

One of the common approaches to alleviating heat-stress in poultry is nutritional manipulation using herbal extracts or their derivatives to maintain the health, welfare, and performance of birds. The present study investigated the protective effect of boldo leaf extract against the harmful effects of cyclic heat stress in two broiler strains (Arbor Acres; AA and Avian-48; AV). Administration of boldo in drinking water was able to restore growth and health traits to nearly normal values. Generally, AA chicks were better able to withstand heat stress and were also more likely to utilize boldo extract than AV chicks. The use of boldo leaf extract in poultry production can assist in mitigating the effect of heat stress, improving the antioxidant defense system, and increasing productivity and profitability.

**Abstract:**

There is increasing interest in the use of natural antioxidant supplements in poultry diets as protection against the adverse effects of heat stress. The potential protective effect of boldo (*Peumus boldus molina*) leaf extract, which have antioxidant activity, were investigated against the harmful effects of heat stress in two broiler strains. Arbor Acres (AA) and Avian-48 (AV) chicks were divided into thermoneutral (TN) and heat stress (HS) groups and treated with 1 g boldo leaf extract/4 L drinking water during the heat stress period. HS reduced growth performance in both strains. The phagocytic index, phagocytic activity, and eosinophil and lymphocytes counts were significantly elevated in TN and HS AV birds but not altered in AA birds. Boldo extract treatment partially eliminated the previous negative impacts of heat stress. AA chicks were better able to withstand HS than AV chicks. Serum concentrations of total lipids and cholesterol were reduced in HS birds of both strains. Malondialdehyde, superoxide dismutase, and glutathione peroxidase levels were elevated but restored with the administration of boldo leaf extract in HS birds of both strains. Economic parameters were negatively affected by HS but restored to values close to those of the control group in boldo-treated HS birds. In conclusion, the administration of boldo leaf extract in drinking water was effective in neutralizing the harmful effects of heat stress on growth performance, blood indices, and economic parameters and improved the antioxidant defense system in heat-stressed birds.

## 1. Introduction

Global warming and climate change adversely affect livestock and poultry production sectors under tropical and subtropical conditions. Chickens are particularly vulnerable to high ambient temperature because of their higher inner body temperature and the lack of sweat glands. Therefore, several physiological disturbances, such as hyperpnea, electrolyte imbalance, rapid pulse, vascular dilatation, endocrine disorders, systemic immune dysregulation, muscle tremors, weakness, and collapse, occur in heat-stressed birds [1,2] that impair their welfare and productivity. Furthermore, high ambient temperature leads to elevated body temperature and, consequently, results in serious tissue damage induced by excessive reactive oxygen species (ROS) [3]. Increased production of ROS is potentially detrimental to the maintenance of homeostasis [4,5]. Moreover, variations in the response of broiler strains and their ability to withstand heat stress have been documented. In general, the heat stress resistance of broiler chickens is low owing to breeding programs constantly selecting for higher growth performance; however, some broiler strains exhibit higher performance and/or higher resistance to climatic changes than others [6,7,8,9]. Therefore, it is essential to implement mitigation strategies that can promote the physiological antioxidant system of these broiler strains to neutralize the excess amounts of generated ROS. Environmental strategies, for example, establishing an intermittent light schedule, increasing ventilation, lowering stocking density, and implementing early feed restriction [10], and nutritional manipulations, for example, use of probiotics [11,12], vitamins [3,13], trace elements [10,13,14,15], and herbal extracts and products [16,17,18], have been considered as common approaches in poultry production. It has been established that the inclusion of herbs and their extracts in poultry diets is generally effective in maintaining their health, welfare, and performance as well as the oxidative stability of their eggs and meat products [16,19,20,21,22].

Boldo (*Peumus boldus molina*) is an abundant native Chilean tree that has been widely used as a medicinal herb to improve hepatic and digestive complaints [23]. Boldo has been used in numerous pharmacopeias as a herbal remedy owing to its high content of polyphenols (flavonoids), alkaloids (boldine), and essential oils [24,25,26,27]. Polyphenols account for as much as approximately 12%–36% of the total solids in the aqueous extract of boldo leaves [23]. Free radical scavenging and lipid peroxidation inhibition activities of the water extract of boldo leaves have been demonstrated [27]. These established characteristics of boldo suggest it as one of the promising herbal plants that could be used to counteract various stressors affecting broiler chicks, especially heat stress. Therefore, the aim of the present study was to investigate the effect of administration of boldo leaf extract in drinking water to mitigate the adverse impacts of heat stress in two broiler strains.

## 2. Materials and Methods

Bird management followed the regulations prescribed by the Animal Care and Ethics Committee (DMU/VetMed-2019-/0040) at the Animal Husbandry and Animal Wealth Development Department, Faculty of Veterinary Medicine, Damanhour University, Damanhour, Egypt.

### 2.1. Experimental Birds, Design, and Management

**Figure 1:**
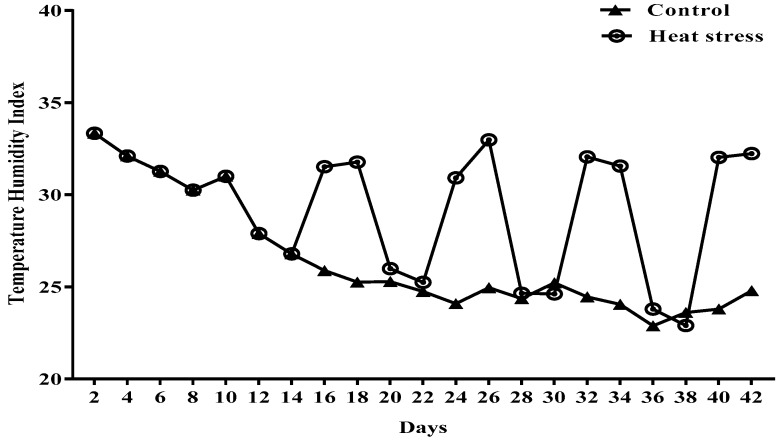
Average temperature humidity index (THI) of thermoneutral and heat stress groups of broilers from 9:00 to 18:00 h during 42 days of the experimental period.

A total of 120 broiler chicks at 14 days of age from two different strains (sixty Arbor Acres (AA) and sixty Avian-48 (AV)) with nearly the same starting body weight (343.3 ± 2.2 g) were used in this study. Each strain was subdivided equally into two experimental groups; one was considered as the control (thermoneutral group, TN), and the other was subjected to high temperature to induce heat stress (HS). Heat-stressed birds were subjected to a temperature of 34 ± 2 °C for 12 h (from 9:00 to 18:00 and then 25 ± 2 °C) for three successive days a week, whereas birds of the TN group were maintained at the common housing temperature (25 ± 2 °C). Relative humidity under both conditions was maintained at 45% ± 3%. The temperature was elevated and maintained constant using electric heaters. Each group was further split into two groups, the control group and the boldo extract treatment group that received 1 g boldo leaf extract/4 L drinking water during the period of heat stress previously described. Three replicates of five birds each were used for the control and boldo-treated groups. Birds were caged in stainless steel batteries with dimensions of 100 × 90 × 45 cm; length × width × height/pen/replicate; equipped with nipple drinkers and manual feeders. During the period of the boldo extract administration, nipple lines were turned upward to prevent birds from consuming water through them and making the birds rely only on manual drinkers that were supplemented with the extract of boldo leaves. Water intake during a certain period was measured by adding the amount of water consumed via nipple drinkers to that consumed via manual drinkers during the same period. Water and feed were offered ad libitum, and all birds were reared under the same managerial and vaccination programs. Manual thermometers and hygrometers placed at the center of the house were used to record and ensure the stability of the indoor temperature and relative humidity four times a day (at 9:00, 12:00, 15:00, and 18:00). The temperature humidity index (THI) was calculated according to the equation described by Tao [2] (THI = 0.85 t_db_ + 0.15 t_wb_, where t_db_ and t_wb_ are dry bulb and wet bulb temperatures, respectively), as depicted in Figure 1. Birds were fed starter and grower diets (Table 1), which were formulated to meet the requirements of the NRC [28]. Arbor Acres and Avian-48 chicks were obtained from commercial hatcheries in Egypt. Boldo extract (the extract of leaves of *Peumus boldus* from the Monimiaceae family) was obtained from Hongda Shaanxi Company in China (Hongda Park, Xizhang Village, Dacheng Town, Sanyuan County, Xianyang City, Shaanxi Province, China).

### 2.2. Growth Performance

Starting from the second week of age, body weight (BW) and feed intake (FI) per replicate were recorded weekly until the 6th week of age. Body weight gain (BWG) was calculated weekly by subtracting the initial body weight at a certain week from the final body weight at the next week. Feed conversion ratio (FCR) was calculated as g feed/g gain per period. Prior to slaughtering, birds were deprived of feed for 12 h and then weighed. Two birds from each replicate were slaughtered, scalded, wet-plucked, and eviscerated and inner organs (liver, heart, spleen, and gizzard) were weighed separately to determine the dressed weight and the dressing percentage. The blood, viscera, lungs, limbs, head, and neck were termed as the offal and discarded. The abdominal fats in the pelvic and abdominal cavity were completely collected from the carcass and then weighed. The carcass was separated into cuts including the breast (breast muscles with the sternum), thigh (average of two thighs weight), shoulder (average of two shoulders weight), and left fillet (the de-skinned left breast muscle on the left side of the sternum), and each was weighed.

### 2.3. Blood Hematology and Biochemistry

Two blood samples were collected in separate labeled centrifuge tubes from the wing vein at 42 days of age from two birds within each replicate. The first tube contained 3.2% sodium citrate solution in order to determine hemoglobin and hematocrit values, white blood cells, red blood cells, and differential leucocytic counts. Phagocytic activity and index were estimated according to the method described by Kawahara et al. [29]. The other tube was left to clot and then centrifuged at 4500× *g* for 15 min. The serum samples were collected and preserved in a deep freezer at (−20 °C) until the time of analysis. Total protein (TP), albumin (ALB), total lipids (TL), triglycerides (TG), and total cholesterol (TC) were spectrophotometrically determined (Spectronic 1201; Milton Roy, Ivyland, PA, USA) using commercial kits from Bio-diagnostic Co., Egypt, according to the manufacturer’s instructions.

Activities of oxidative stress biomarkers, including malondialdehyde (MDA), glutathione peroxidase (GPx), and superoxide dismutase (SOD), were assessed using the ELISA Kit from Quanti Chrom^TM^, BioAssay Systems, USA and Cayman Chemical Company, USA.

### 2.4. Economic Efficiency

Feed cost (US $/kg feed) was calculated by multiplying the total feed intake per bird by the cost of one kg feed (0.36 US $/kg feed). The average cost of feed/kg body weight (US $/kg gain) was calculated as feed cost (US $/kg feed) × feed intake per bird (kg)/weight gain per bird (kg). The cost of the boldo extract supplementation (0.02 US $/bird) was included in the total costs of the treatment groups. The other expenditure including the cost of day-old chicks, housing, labor, drugs, disinfectant, vaccines, veterinary supervision, and depreciation cost were common to all groups. Total costs were calculated by summing all fixed costs and variable costs. The net return was obtained by the difference between the total return, considering the average value of the bird (1.45 US $/kg live body weight) and the feeding cost for each group. Benefit/cost ratio (B/C ratio) was calculated as total return/total costs.

### 2.5. Statistical Analysis

Data were analyzed using the Statistical Analysis System (SAS, 2002), three-way analysis of variance, and the general linear model procedure. The effect of fixed factors, broiler strain, heat stress, and administration of boldo extract, and their interaction were estimated. Tukey’s multiple comparison test was used to identify the presence of significance (*p* < 0.05) among multiple means. To ensure that the results from the three replicates used were sufficient and the dispersion of the data around the means was unique and within the accepted limits, the coefficient of variation (C.V.%) was calculated as

(1) $$C.V.\%=(\mathrm{SD}/\bar{X})\times 100$$

where SD is the standard deviation, and $\bar{X}$ is the sample mean. C.V.% values are listed in all tables; it was noted that all C.V.% values were within the acceptable limits.

## 3. Results

### 3.1. Growth Performance

The results of live body weight and body weight gain as affected by broiler strain, heat stress, or treatment with boldo extract are presented in Table 2. Significant differences between final BW and BWG of AA and AV broiler chicks were observed in response to HS or the treatment with boldo. Generally, AA birds were superior in their growth in comparison to AV birds. Final BW and BWG were higher in HS AA birds (2180 and 1610 g, respectively) than those of HS AV birds (2040.5 and 1459.8 g, respectively) and in TN AA than in TN AV birds (2285.4 and 1698.9 vs. 2156.2 and 1567.7 g, respectively). Regardless of broiler strain, boldo supplementation significantly or numerically enhanced BW and BWG values during all experimental periods either under HS or TN conditions, respectively. It is worth noting that AA birds benefited from boldo treatment more than AV birds. Values of BW and BWG increased by about 4.8% and 6.3%, respectively, under the TN environment and 5.1% and 7.6%, respectively, under the HS environment.

In general, AA birds consumed more feed than AV birds during the experimental period (Table 3). In each strain, neither boldo treatment nor HS significantly affected FI during the initial experimental periods (2–3, 3–4, and 4–5 weeks of age). AV birds exposed to HS consumed less feed during the final experimental period (5–6 weeks of age) and the overall duration of the experiment (2–6 weeks of age) regardless of boldo administration. Furthermore, treatment with boldo reduced FCR in both strains at 3–4 weeks of age. No significant alterations among the experimental groups were detected at 4–5 weeks of age (Table 3). However, during the last experimental period (5–6 weeks of age), boldo-treated AV birds recorded the lowest FCR value in comparison to that of boldo-treated AA birds under TN and HS conditions. Overall, FCR improved in AA birds treated with boldo and reared under the TN environment, whereas no significant differences were observed among the remaining groups. Results presented in Table 3 also revealed no significant alteration in water intake in the broiler strains regardless of the treatment with boldo or HS during all the experimental periods. However, an elevation in water intake was observed in heat-stressed AV and AA birds either treated or not with boldo.

Relative weights of the heart, spleen, thigh, left fillet, and abdominal fat were not affected by the interaction among broiler strains, ambient temperature, and boldo treatment. However, dressing percentage and relative weights of the liver, gizzard, breast, and shoulder were affected (Table 4). Dressing and liver percentages were significantly reduced in HS AV birds compared to those of HS AA birds. The highest dressing% value was observed in AV chicks treated with boldo and reared under the TN environment, whereas the lowest value was recorded in HS AV birds not treated with boldo extract. Moreover, the breast% was significantly reduced in AV birds exposed to heat stress compared to that in TN birds. The highest values of breast% were observed in TN AV and AA birds treated with boldo extract.

### 3.2. Hematological Parameters

The hematological parameters were greatly affected by broiler strain, heat stress, and boldo treatment (Table 5). Total counts of erythrocytes and leucocytes and hematocrit and hemoglobin values were not altered by any of the fixed factors and their interactions. Phagocytic index, phagocytic activity, and counts of eosinophil and lymphocytes were significantly reduced in TN and HS AV birds that were not treated with boldo. These parameters were not altered in the corresponding AA birds. Counts of heterophils, basophils, and monocytes did not differ in HS AA and AV birds, except for the monocytes count, which was lower in HS AA chickens than in the TN AA birds. Moreover, treatment with boldo did not affect the aforementioned parameters in AV birds reared under TN or HS. Boldo-treated AA birds reared under the same conditions showed a significant increase in heterophils count and a decrease in monocytes count, whereas counts of basophil were not affected.

### 3.3. Serum Biochemical Parameters

Serum levels of TP, ALB, A/G ratio, and TG were not affected by the interaction among experimental factors (Table 6). The ALB level tended to increase in both strains treated with boldo either under TN or HS conditions. The GLO level was significantly reduced in HS AV birds and elevated in HS AA chicks compared to TN birds of both strains not treated with boldo. Moreover, exposing AV birds to heat stress decreased serum concentrations of TL and TC compared to that in TN birds, whereas they increased in HS AA birds compared to that in TN birds. Treatment with boldo extract in HS or TN AV birds did not significantly alter these parameters but reduced their levels only in HS AA birds compared to that in the untreated HS birds. Levels of oxidative stress biomarkers (MDA, GPx, and SOD) were increased in AV and AA birds exposed to heat stress compared to those reared under the TN condition (Table 7). All the previous parameters were significantly reduced in both strains exposed to heat stress and treated with boldo extract compared to that in TN birds. Moreover, treatment with boldo extract decreased serum MDA content and GPx activity in TN AA birds and the value of GPx only in TN AV birds.

### 3.4. Economic Efficiency

As presented in Table 8, feed cost, feed cost/gain, and total costs were not altered among the experimental factors and their interactions. Birds of both strains exposed to HS and not treated with boldo extract showed significant deterioration in total return, net return, and B/C ratio (for AV birds only) compared to the control birds. However, these parameters were significantly improved in HS AV and AA birds treated with boldo leaf extract. TN birds of AA treated with boldo recorded the highest values of the aforementioned parameters followed by AV birds reared under the same conditions.

## 4. Discussion

Results of the present investigation revealed that the administration of boldo extract in drinking water was effective in promoting growth of birds reared under the TN condition and partially enhanced the ability to recover weight loss in HS birds. Growth of AA birds was less affected than that of AV birds by HS. Furthermore, the exposure to HS significantly reduced FI of birds regardless of the treatment with boldo extract; whereas FCR was not significantly affected. Broiler strain affected FI; AV birds consumed less feed than AA birds in general. Water intake was not significantly altered by the interaction among broiler strain × heat stress × boldo treatments with an increase in water consumption by heat-stressed birds of both strains. Our results are consistent with those of previous studies [30,31,32]. The adverse effect of HS on broiler growth performance can be attributed to the mucosal epithelial damage caused by HS that impairs nutrients absorption. In addition, heat-stressed birds expend more energy to adapt to HS conditions, leading to the reduction in their growth performance as less energy is used for growth [31]. Another possible explanation for the reduction in growth and FI is that under the HS condition, poor appetite and lower FI were observed; these are considered as a defense mechanism to reduce the increment in body heat of broilers [31]. Furthermore, the impaired growth rate in heat-stressed birds might also be attributed to changes in the blood circulation pattern [33]. Under high ambient temperature, blood supply to the peripheral organs increases to promote the dissipation of inner heat to the environment. Consequently, blood flow to the gut is reduced, leading to a leaky gut, greatly deprived in nutrients and oxygen. Under such circumstances, disturbance in the intestinal ecosystem and reduction in bacterial substances production occurred [33], which in turn reduced the growth of birds. Moreover, the decreased amount of bacterial substances such as lipopolysaccharides stimulates the production of pro-inflammatory cytokines, for example, TNF-Y, IL-1α, IL-1β, and IL-6, which activates T and B lymphocytes in order to eliminate the damaged tissue. The increase in water consumption of heat-stressed birds can be attributed to the important role of water in maintaining thermoregulatory balance, as birds under heat stress conditions lose a high amount of water through evaporative cooling via the respiratory tract as a means of achieving effective thermal regulation. Supplementation of phytogenic additives (FA), such as boldo extract, is able to reverse the deleterious impacts of heat stress through promoting the growth of healthier microbes [34], reducing the creation of growth-depressing microbial substances, such as biogenic amines and ammonia [35,36], and increasing the availability of nutrients to the host [37]. Furthermore, it has been reported that essential oils of FA might be efficient in controlling the intestinal mucosa inflammation evidenced by the reduction in the number of intra-epithelial leucocytes of the intestinal mucosa of pigs [38]. The control of intestinal inflammation should spare nutrients for absorption [39,40]. Additionally, FA can enhance the production of digestive enzymes and manipulate the intestinal microflora [41,42]. These previously demonstrated cumulative effects of boldo may explain the improvement in the growth performance of heat-stressed birds treated with boldo leaf extract. Moreover, AA chicks were better able to withstand heat stress as these birds showed higher BW, BWG, and FI than AV chickens. Heat-stressed boldo-treated AA chicks exhibited a higher growth rate than even heat-stressed AV birds treated or not with boldo extract. Our results are consistent with those reported by Hascik et al. [6] and Abdo et al. [9].

Heat stress decreased dressing percentage and relative weights of the liver and breast in AV birds compared to those of AA birds. Boldo treatment restored values of these parameters near to the values in TN birds and increased their values in the TN birds. Consistent with our results, Shim et al. [43] and Plavnik and Yahav [44] reported that the relative liver weight was lower in heat-stressed chicks as a result of decreased metabolic needs. Song et al. [31] reported that the proportion of breast muscle as a percentage of BW was reduced by cyclic heat stress. Relative weight of the pectoralis major of birds reared under 32 °C was lower than that of those reared under 22 °C [45]. The authors attributed this reduction in muscle weight to the elevation of protein synthesis susceptibility than to proteolysis at 32 °C, which thereby lowered the deposition of muscle protein. The amelioration effects of FA on carcass traits of heat-stressed birds have been reported [32,46,47,48]. Possible explanations for the heat stress mitigation effect of FA may be their ability to boost the antioxidant defense system, immune response, digestive enzyme secretion as well as stimulate the appetite and feed intake [32,49]. Moreover, the effects of heat exposure on abdominal fat have been controversial among researchers for a long time. Consistent with our results, Smith and Teeter [50] and Fisher [51] reported a significant reduction in fat content in heat-stressed birds, whereas an elevation in fat deposition was observed by Baziz et al. [52] and Geraert et al. [53]. The reduced fat pad size observed in boldo-treated birds compared to that in heat-stressed birds suggested the reduction in fat deposition through increasing lipolysis and/or inhibition of the lipogenesis process [54].

Regarding hematological observations, our results indicated no interaction effect among heat stress, boldo treatment, and chicken strain on values of blood erythrocytes, leucocytes, hemoglobin, and hematocrit. These results are in agreement with those of Tamzil et al. [55], who observed that these parameters were not altered in normal kampong, Arabic, and commercial chickens compared to that in birds exposed to acute heat stress. However, heat stress significantly affected the phagocytic index and activity and proportional count of leucocytes. It has been reported that heat stress led to increased counts of basophils, heterophils, and lymphocytes but did not affect the eosinophil count [55,56]. These disturbances in leucocytes counts may be because of the adverse effect of heat burden on lipid peroxidation, inflammatory response, and tissue damage that trigger the production of phagocytic leucocytes to engulf damaged cells and foreign pathogens [57]. Moreover, the increased heterophil percentage and depressed lymphocyte count may be attributed to the elevation in glucocorticoid and corticosterone secretion under high ambient temperature conditions [55]. Administration of boldo extract in drinking water was able to balance the heat burden and restore the leucocytes count close to the values of TN birds because of the ability of its bioactive substances to control intestinal mucosa inflammation and inhibit enteric pathogens, which subsequently down-regulated leukocyte synthesis.

Serum TP, A/G ratio, and TG levels were not affected by the fixed factors or their interactions. However, the ALB level increased in both strains treated with boldo either under TN or HS conditions but was not affected by the other fixed factors or their interactions. Our results agree with the previous investigations, which used menthol, anethol, and eugenol sources of FA [58,59,60]. In contrast to our findings, Reis et al. [61] reported a significant reduction in serum concentration of TP and GLO in broilers fed FA containing diets. The authors linked this reduction in serum protein levels to the lower immune response evidenced by lower lymphocyte count. Moreover, serum concentrations of TL and TC were significantly lower in chicks exposed to heat stress than in TN birds. These results are consistent with those of Shim et al. [43] and Takahashi and Jensen [62]. However, the serum level of cholesterol tended to increase, especially in AA-stressed birds. Increase in the cholesterol level may be caused by the lipolysis of body lipids in order to compensate for the energy required resulting from the decrease in feed intake [63]. However, administration of boldo extract in drinking water induced a reversal of these factors to levels of the control, thereby implying that boldo extract increased lipogenesis in the liver of heat-stressed chickens.

It is well-documented that heat stress augments the generation of free radicals, which increases lipid peroxidation [1,5,8,12]. As an indicator of lipid peroxidation, MDA concentration increased in the blood of heat-stressed birds concomitantly with an elevation in the activities of antioxidant enzymes (SOD and GPx). These increases in antioxidant enzyme activities during the period of non-damaging exercise have been considered as a physiological response to protect living cells from oxidative stress [64,65,66]. In the present study, both broiler strains exposed to high ambient temperature showed higher serum levels of MDA, GPx, and SOD. However, the treatment with boldo reduced the values of these parameters. The similar response of the antioxidant defense system of both strains to heat stress may be attributed to the large differences in gene expression of these enzymes. Abdo et al. [9] reported that gene expressions of SOD and catalase in the liver of heat-stressed Ross and Cobb chickens were not significantly altered. Furthermore, the strong free radical scavenging and antioxidant activity of boldo leaves could be attributed to their high content of boldine and catechin [67]. Boldine has been reported as the principal antioxidant alkaloid fraction [68], whereas, the total antioxidant activity of catechin, the main flavonoid compound, is estimated to be 60.9% [23,69]. These active compounds compensate in scavenging and neutralizing the excess of free radicals that subsequently boosts the antioxidant system of heat-stressed birds and reduces the levels of the studied oxidative stress biomarkers to the normal level [70,71,72,73,74].

The enhancement of economic efficiency in boldo-treated groups may be attributed to the ability of the boldo leaf extract to increase broiler growth performance, improve feed efficiency, and reduce bird mortality by stimulating their immune system. Moreover, AA chicks were more responsive to the utilization of boldo extract and exhibited higher profitability after treatment than AV birds. Previous investigations attributed the alterations in profitability in broiler production to the variations in growth rate and the cost of one-day-old chicks, drugs, labor, feed, veterinary services, and the sale price of birds [75,76]. Our results are consistent with those reported in earlier studies [32,77,78].

## 5. Conclusions

Administration of boldo leaf extract as a natural antioxidant supplement in broiler drinking water at the investigated level was able to partially protect the birds from the adverse effects of heat stress. Treatment with boldo extract alleviated the marked reduction in growth performance, antioxidative status, and economic return of heat-stressed birds. The AA chicks were less affected by heat stress and more likely to utilize the boldo extract than AV chicks. Limited research has been performed to study the effect of boldo leaves on poultry performance; therefore, further investigations are required to determine the optimal dose and best route of administration.

## Figures and Tables

**Table 1:** Ingredients and calculated chemical composition of the basal diets.

Ingredients (%)	Starter (1–21 days)	Grower (22–42 days)
Yellow corn	54.03	58.98
Soybean meal (44%)	34.50	29.50
Corn germ (62%)	5.50	5.50
Soybean oil	1.80	2.30
Limestone	1.08	0.95
Di-Calcium Phosphate	2.00	1.75
Premix ^1^	0.30	0.30
NaCl	0.30	0.30
L-lysine	0.29	0.24
DL-Methionine	0.20	0.18
Calculated composition ^2^ (%)		
Metabolizable energy (ME, kcal kg^−1^)	3001	3180
Crude protein	23.12	20.99
Calcium	0.99	0.89
Potassium	0.54	0.52
Phosphorus (available)	0.51	0.46
Digestible methio + Cys	0.93	0.89
Digestible methionine	0.59	0.52
Digestible lysine	1.43	1.24
Digestible arginine	1.25	1.07
Digestible tryptophan	0.19	0.17

^1^ Each kg of diet provides: Vit. A: 12,000 IU, Vit. D_3_: 5000 IU, Vit. E: 130.0 mg, Vit. K_3_: 3.61 mg, Vit. B_1_: 3.0 mg, Vit. B_2_: 8.0 mg, Vit. B_6_: 4.95 mg, Vit. B_12_: 0.17 mg, Niacin: 60.0 mg, Folic acid: 2.08 mg, D-Biotin: 200.0 mg, calcium D-Pantothenate: 18.33 mg, Copper: 80.0 mg, Iodine: 2.0 mg, Selenium: 150.0 mg, Iron: 80.0 mg, Manganese: 100.0 mg, Zinc: 80.0 mg, Cobalt: 500.0 mg. ^2^ Calculated according to NRC [28].

**Table 2:** Effect of broiler strain, heat stress, and boldo supplementation on body weight and weight gain of broilers.

Heat Stress	Boldo	Live Body Weight (g)					Body Weight Gain (g/bird/week)				
		2 Weeks	3 Weeks	4 Weeks	5 Weeks	6 Weeks	2–3 Weeks	3–4 Weeks	4–5 Weeks	5–6 Weeks	2–6 Weeks
**Avian-48**											
TN	−	344.7	588.5 ^bc^	1133.1 ^e^	1660.7 ^e^	2156.2 ^e^	243.8	544.6 ^e^	527.6 ^abcd^	495.5 ^ab^	1811.5 ^d^
TN	+	341.3	592.5 ^b^	1184.5 ^cd^	1704.5 ^d^	2220.4 ^c^	251.2	592.0 ^cd^	520.0 ^bcd^	515.9 ^a^	1879.1 ^c^
HS	−	343.7	580.7 ^d^	1141.9 ^de^	1615.8 ^f^	2040.5 ^g^	237.0	561.2 ^de^	473.9 ^d^	424.7 ^d^	1696.8 ^f^
HS	+	342.0	592.8 ^b^	1159.5 ^cde^	1672.5 ^e^	2107.0 ^f^	250.8	566.7 ^cde^	513.0 ^cd^	434.5 ^d^	1765 ^e^
**Arbor Acres**											
TN	−	341.7	586.5 ^c^	1234.1 ^b^	1799.5 ^b^	2285.4 ^b^	244.8	647.6 ^b^	565.4 ^abc^	485.9 ^bc^	1943.7 ^b^
TN	+	345.7	597.8 ^a^	1302.6 ^a^	1860.6 ^a^	2327.9 ^a^	252.1	704.8 ^a^	558.0 ^abc^	467.3 ^c^	1982.2 ^a^
HS	−	344.3	570.0 ^e^	1168.8 ^cde^	1751.4 ^c^	2180.0 ^d^	225.7	598.8 ^cd^	582.6 ^a^	428.6 ^d^	1835.7 ^c^
HS	+	343.0	585.1 ^c^	1194.0 ^bc^	1769.1 ^c^	2214.2 ^c^	242.1	608.9 ^bc^	575.1 ^ab^	445.1 ^d^	1871.2 ^c^
***p* Value**											
Strain (S)		0.666	0.063	>0.001	>0.001	>0.001	0.868	>0.001	>0.001	0.213	>0.001
Heat stress (HS)		0.962	>0.001	>0.001	>0.001	>0.001	0.147	<0.001	0.610	>0.001	>0.001
Boldo (B)		0.737	>0.001	>0.001	0.011	0.014	0.078	0.010	0.753	0.390	>0.001
S × HS		0.962	>0.001	>0.001	>0.001	>0.001	0.125	>0.001	>0.001	>0.001	>0.001
S × B		0.278	>0.001	>0.001	>0.001	>0.001	0.468	>0.001	>0.001	0.390	>0.001
HS × B		0.599	>0.001	>0.001	>0.001	>0.001	0.159	>0.001	0.822	>0.001	>0.001
S × HS × B		0.321	>0.001	>0.001	>0.001	>0.001	0.749	>0.001	>0.001	>0.001	>0.001
SEM		0.774	1.032	7.038	8.935	10.066	0.663	7.394	7.333	4.408	9.767
C.V.%		1.12	1.57	5.76	4.62	4.10	8.21	10.96	12.15	8.52	5.44

TN: thermoneutral temperature; HS: heat stress; SEM: Standard error of mean; C.V.%: coefficient of variation. Data presented as means and means in the same row with no superscript letters after them or with a common superscript letter following them are not significantly different (*p* < 0.05).

**Table 3:** Effect of broiler strain, heat stress, and boldo supplementation on feed intake and feed conversion ratio of broilers.

Heat Stress	Boldo	Water Intake (mL/(Bird·Period))					Feed Intake (g/(Bird·Period))					Feed Conversion Ratio (g Feed/g Gain)				
		2–3 Weeks	3–4 Weeks	4–5 Weeks	5–6 Weeks	2–6 Weeks	2–3 Weeks	3–4 Weeks	4–5 Weeks	5–6 Weeks	2–6 Weeks	2–3 Weeks	3–4 Weeks	4–5 Weeks	5–6 Weeks	2–6 Weeks
**Avian-48**																
TN	−	1113.0	1495.0	1770.7	2188.3	6567.0	333.9	754.0 ^b^	902.5 ^a^	1132.5 ^a^	3122.9 ^b^	1.37	1.39 ^ab^	1.71 ^ab^	2.29 ^c^	1.72 ^ab^
TN	+	1113.7	1509.7	1748.3	2219.3	6591.0	327.6	751.0 ^b^	880.0 ^b^	1115.0 ^a^	3073.6 ^bc^	1.30	1.27 ^c^	1.69 ^ab^	2.16 ^d^	1.64 ^bc^
HS	−	1285.3	1901.3	2302.3	3034.0	8523.0	319.8	744.0 ^b^	872.5 ^b^	1052.5 ^b^	2988.8 ^cde^	1.35	1.33 ^bc^	1.84 ^a^	2.48 ^a^	1.76 ^a^
HS	+	1262.3	1870.7	2272.3	2950.3	8358.7	323.4	749.5 ^b^	882.5 ^b^	1007.5 ^b^	2962.9 ^e^	1.29	1.32 ^bc^	1.72 ^ab^	2.32 ^c^	1.68 ^ab^
**Arbor Acres**																
TN	−	1154.0	1589.0	1803.0	2112.3	6658.3	339.3	882.5 ^a^	917.5 ^a^	1095.0 ^b^	3234.3 ^a^	1.39	1.36 ^ab^	1.62 ^ab^	2.25 ^cd^	1.66 ^bc^
TN	+	1168.0	1583.0	1776.3	2148.3	6671.6	340.1	871.5 ^a^	917.5 ^a^	1092.5 ^b^	3221.6 ^a^	1.35	1.24 ^c^	1.64 ^ab^	2.34 ^bc^	1.63 ^c^
HS	−	1335.0	2006.0	2319.3	3003.3	8663.7	311.9	852.5 ^a^	917.5 ^a^	1080.0 ^b^	3161.9 ^a^	1.38	1.42 ^a^	1.58 ^b^	2.52 ^a^	1.72 ^ab^
HS	+	1303.3	1992.0	2273.7	2964.7	8533.7	312.7	847.5 ^a^	905.0 ^a^	1082.5 ^b^	3147.7 ^ab^	1.29	1.39 ^ab^	1.57 ^b^	2.43 ^ab^	1.68 ^ab^
***p* Value**																
Strain (S)		>0.001	>0.001	0.485	0.388	0.121	0.682	>0.001	>0.001	0.205	>0.001	0.853	0.084	0.041	0.070	0.201
Heat stress (HS)		>0.001	>0.001	>0.001	>0.001	>0.001	0.059	0.214	0.010	>0.001	>0.001	0.267	0.067	0.953	>0.001	0.113
Boldo (B)		0.078	0.138	0.274	0.767	0.401	0.347	0.790	0.135	0.065	0.216	0.337	0.072	0.247	0.067	0.229
S × HS		0.868	0.022	0.708	0.488	0.636	0.914	>0.001	>0.001	>0.001	>0.001	0.652	0.014	0.023	>0.001	>0.001
S × B		0.916	0.864	0.858	0.789	0.938	0.723	>0.001	>0.001	0.063	>0.001	0.761	>0.001	0.051	0.013	>0.001
HS × B		0.011	0.034	0.811	0.319	0.282	0.331	0.640	0.082	>0.001	>0.001	0.444	>0.001	0.682	>0.001	>0.001
S × HS × B		0.348	0.125	0.919	0.831	0.882	0.155	>0.001	>0.001	>0.001	>0.001	0.269	>0.001	0.035	>0.001	>0.001
SEM		17.69	42.91	55.29	88.05	201.0	5.258	6.511	2.056	4.125	10.16	0.023	0.012	0.025	0.022	0.014
C.V.%		4.65	5.64	6.51	5.64	7.56	3.56	7.22	2.05	3.40	3.25	4.23	8.73	12.04	7.08	3.21

TN: thermoneutral temperature; HS: heat stress; SEM: standard error of mean; C.V.%: coefficient of variation. Data presented as means and means in the same column with no superscript letters after them or with a common superscript letter following them are not significantly different (*p* < 0.05).

**Table 4:** Effect of broiler strain, heat stress, and boldo supplementation on carcass traits as % of slaughter weight of broilers.

Heat Stress	Boldo	Dressing	Liver	Gizzard	Heart	Spleen	Fat	Breast	Thigh	Shoulder	Left Fillet
**Avian-48**											
TN	−	71.34 ^abc^	4.20 ^ab^	2.92 ^ab^	0.87	0.20	1.67 ^ab^	27.18 ^ab^	16.62	4.26 ^ab^	11.24
TN	+	72.70 ^a^	4.43 ^a^	3.07 ^a^	0.89	0.20	1.59 ^b^	27.87 ^a^	16.76	4.37 ^ab^	11.31
HS	−	68.58 ^d^	3.82 ^c^	2.92 ^ab^	0.85	0.20	2.01 ^a^	24.54 ^c^	15.35	3.75 ^b^	10.15
HS	+	70.71 ^bc^	4.18 ^ab^	2.89 ^ab^	0.86	0.20	1.79 ^ab^	27.32 ^ab^	16.10	4.28 ^ab^	11.16
**Arbor Acres**											
TN	−	70.05 ^c^	4.02 ^bc^	2.71 ^b^	0.87	0.20	1.72 ^ab^	25.77 ^bc^	16.43	4.03 ^ab^	10.63
TN	+	72.03 ^ab^	4.28 ^ab^	3.04 ^a^	0.90	0.20	1.66 ^ab^	27.72 ^a^	16.29	4.42 ^a^	11.50
HS	−	70.08 ^c^	4.03 ^bc^	2.74 ^b^	0.87	0.22	1.70 ^ab^	25.71 ^bc^	16.12	4.22 ^ab^	10.58
HS	+	71.16 ^bc^	3.98 ^bc^	3.00 ^a^	0.88	0.19	1.90 ^ab^	26.58 ^ab^	15.38	4.57 ^a^	11.29
***p* Value**											
Strain (S)		0.983	0.279	0.226	0.565	0.739	0.803	0.459	0.678	0.282	0.907
Heat stress (HS)		>0.001	>0.001	0.391	0.353	0.971	0.024	>0.001	0.030	0.634	0.140
Boldo (B)		>0.001	0.015	>0.001	0.244	0.686	0.653	>0.001	0.993	0.020	0.016
S × HS		0.011	0.028	0.492	0.541	0.733	0.329	0.043	0.637	0.098	0.344
S × B		>0.001	0.046	0.041	0.790	0.552	0.202	>0.001	0.233	0.861	0.633
HS × B		>0.001	>0.001	0.021	0.701	0.608	0.731	>0.001	0.989	0.491	0.456
S × HS × B		>0.001	>0.001	0.023	0.955	0.287	0.056	0.030	0.411	0.042	0.299
SEM		0.211	0.043	0.035	0.001	0.001	0.047	0.214	0.185	0.074	0.132
C.V.%		2.69	8.84	9.63	8.53	16.94	21.86	7.25	10.14	15.10	10.86

TN: thermoneutral temperature; HS: heat stress; SEM: standard error of mean; C.V.%: coefficient of variation. Data presented as means and means in the same column with no superscript letters after them or with a common superscript letter following them are not significantly different (*p* < 0.05).

**Table 5:** Effect of broiler strain, heat stress, and boldo supplementation on hematological parameters of broilers.

Heat Stress	Boldo	Leucocytes, 10^3^/mm^3^	Erythrocytes, 10^6^/mm^3^	Hematocrit, %	Hemoglobin, %	Phagocytic Index	Phagocytic Activity	Eosinophils, %	Lymphocyte, %	Heterophils, %	Basophils, %	Monocyte, %
**Avian-48**												
TN	-	23.87	3.18	29.14	14.06	1.42 ^b^	15.14 ^b^	8.10 ^c^	34.96 ^b^	23.34 ^bc^	1.06 ^bc^	5.36 ^bc^
TN	+	23.86	3.21	29.26	14.09	1.72 ^a^	16.40 ^a^	8.54 ^a^	36.20 ^a^	23.22 ^c^	1.07 ^abc^	5.37 ^bc^
HS	-	23.79	3.16	29.22	14.10	1.42 ^b^	14.80 ^b^	8.16 ^c^	34.38 ^b^	23.38 ^abc^	1.03 ^c^	5.16 ^c^
HS	+	23.79	3.23	29.17	14.32	1.72 ^a^	17.00 ^a^	8.38 ^ab^	36.36 ^a^	23.72 ^a^	1.10 ^ab^	5.44 ^b^
**Arbor Acres**												
TN	-	23.86	3.26	29.14	14.08	1.72 ^a^	17.10 ^a^	8.56 ^a^	36.68 ^a^	23.26 ^c^	1.08 ^abc^	5.68 ^a^
TN	+	23.79	3.21	29.16	14.02	1.70 ^a^	16.30 ^a^	8.50 ^a^	36.12 ^a^	23.66 ^ab^	1.11 ^a^	5.34 ^bc^
HS	-	23.82	3.19	29.14	14.22	1.60 ^a^	16.20 ^a^	8.28 ^bc^	36.02 ^a^	23.22 ^c^	1.08 ^abc^	5.42 ^b^
HS	+	24.08	3.22	29.08	14.18	1.62 ^a^	16.40 ^a^	8.46 ^ab^	36.00 ^a^	23.54 ^ab^	1.10 ^ab^	5.48 ^ab^
***p* Value**												
Strain (S)		0.435	0.155	0.475	0.379	0.012	>0.001	>0.001	>0.001	0.950	0.133	0.011
Heat stress (HS)		0.704	0.428	0.827	0.024	0.015	0.498	0.043	0.132	0.264	0.920	0.223
Boldo (B)		0.539	0.170	0.930	0.220	>0.001	>0.001	>0.001	>0.001	0.016	>0.001	0.934
S × HS		0.164	0.464	0.840	0.494	0.154	0.184	0.030	0.051	0.041	0.847	0.938
S × B		0.487	0.081	0.779	0.020	>0.001	>0.001	0.011	>0.001	0.042	>0.001	0.010
HS × B		0.220	0.079	0.513	0.615	0.013	0.010	0.016	0.054	0.043	0.012	>0.001
S × HS × B		0.276	0.586	0.825	0.709	>0.001	>0.001	0.019	>0.001	0.020	0.014	>0.001
SEM		0.032	0.010	0.042	0.031	0.025	0.153	0.034	0.143	0.016	0.013	0.035
C.V.%		0.92	1.77	0.96	1.20	9.77	5.90	2.49	2.57	1.28	3.47	3.78

TN: thermoneutral temperature; HS: heat stress; SEM: standard error of mean; C.V.%: coefficient of variation. Data presented as means and means in the same column with no superscript letters after them or with a common superscript letter following them are not significantly different (*p* < 0.05).

**Table 6:** Effect of broiler strain, heat stress, and boldo supplementation on blood metabolites of broilers.

Heat Stress	Boldo	Albumin (g/dL)	Globulin (g/dL)	AG Ratio	Total Protein (g/dL)	Total Lipid (mg/dL)	Triglycerides (mg/dL)	Cholesterol (mg/dL)
**Avian-48**								
TN	-	1.75	1.76 ^a^	0.99	3.51	677.04 ^a^	157.13	181.38 ^a^
TN	+	1.88	1.67 ^ab^	1.12	3.55	607.18 ^ab^	146.18	161.63 ^a^
HS	-	1.66	1.49 ^b^	1.11	3.15	461.54 ^c^	127.79	137.90 ^b^
HS	+	1.84	1.61 ^ab^	1.17	3.45	455.37 ^c^	128.90	133.53 ^b^
**Arbor Acres**								
TN	−	1.80	1.62 ^ab^	1.12	3.42	427.33 ^c^	123.16	131.36 ^b^
TN	+	1.90	1.68 ^ab^	1.14	3.59	582.52 ^b^	142.87	161.14 ^a^
HS	−	1.75	1.76 ^a^	0.99	3.51	677.04 ^a^	157.13	181.38 ^a^
HS	+	1.84	1.61 ^ab^	1.17	3.45	455.37 ^c^	128.90	133.53 ^b^
***p* Value**								
Strain (S)		0.331	0.493	0.913	0.241	0.455	0.797	0.738
Heat stress (HS)		0.175	0.227	0.754	0.065	>0.001	0.386	0.025
Boldo (B)		0.013	0.780	0.068	0.099	0.071	0.540	0.056
S × HS		0.982	0.061	0.180	0.136	>0.001	0.161	>0.001
S × B		0.522	0.583	0.957	0.383	0.908	0.963	0.778
HS × B		0.817	0.995	0.709	0.887	>0.001	0.240	>0.001
S × HS × B		0.730	0.053	0.230	0.078	>0.001	0.165	>0.001
SEM		0.023	0.033	0.025	0.046	15.03	3.921	4.978
C.V.%		8.05	10.58	13.94	6.56	14.97	12.16	16.44

TN: thermoneutral temperature; HS: heat stress; SEM: standard error of mean; C.V.%: coefficient of variation. Data presented as means and means in the same column with no superscript letters after them or with a common superscript letter following them are not significantly different (*p* < 0.05).

**Table 7:** Effect of broiler strain, heat stress, and boldo supplementation on blood oxidative status of broilers.

Heat Stress	Boldo	MDA (nmoles/mL)	GPx (U/gHb)	SOD (U/gHb)
**Avian-48**				
TN	−	1.64 ^cd^	17.60 ^ef^	48.80 ^d^
TN	+	1.56 ^cd^	15.60 ^h^	47.40 ^d^
HS	−	2.70 ^a^	25.80 ^b^	89.60 ^a^
HS	+	2.34 ^b^	21.40 ^d^	73.20 ^c^
**Arbor acres**				
TN	−	1.68 ^c^	18.00 ^e^	49.40 ^d^
TN	+	1.52 ^d^	16.40 ^gh^	47.00 ^d^
HS	−	2.64 ^a^	27.60 ^a^	90.60 ^a^
HS	+	2.40 ^b^	23.00 ^c^	78.80 ^b^
***p* Value**				
Strain (S)		0.991	0.034	0.071
Heat stress (HS)		>0.001	>0.001	>0.001
Boldo (B)		>0.001	>0.001	>0.001
S × HS		>0.001	>0.001	0.050
S × B		>0.001	>0.001	>0.001
HS × B		0.013	>0.001	>0.001
S × HS × B		0.030	>0.001	>0.001
SEM		0.078	0.693	1.946
C.V.%		13.77	11.13	15.41

MDA: malondialdehyde; GPx: glutathione peroxidase; SOD: superoxide dismutase; TN: thermoneutral temperature; HS: heat stress; SEM: standard error of mean; C.V.%: coefficient of variation. Data presented as means and means in the same column with no superscript letters after them or with a common superscript letter following them are not significantly different (*p* < 0.05).

**Table 8:** Effect of broiler strain, heat stress, and boldo supplementation on the economic parameters.

Heat Stress	Boldo	Feed Cost (US $/Bird)	Feed Cost/kg Gain (US $/kg)	Total Cost (US $/Bird)	Total Return (US $/Bird)	Net Return (US $/Bird)	B/C Ratio (%)
**Avian-48**							
TN	-	1.00	0.64	1.78	3.13 ^c^	2.12 ^e^	175.84 ^c^
TN	+	0.98	0.60	1.79	3.22 ^b^	2.23 ^c^	179.88 ^b^
HS	-	0.96	0.66	1.74	2.96 ^e^	2.00 ^f^	170.11 ^d^
HS	+	0.95	0.62	1.76	3.05 ^d^	2.10 ^e^	173.29 ^c^
**Arbor Acres**							
TN	-	1.04	0.61	1.82	3.31 ^a^	2.27 ^b^	181.86 ^a^
TN	+	1.03	0.60	1.84	3.37 ^a^	2.34 ^a^	183.15 ^a^
HS	-	1.02	0.63	1.80	3.16 ^c^	2.13 ^e^	175.55 ^c^
HS	+	1.02	0.62	1.83	3.21 ^b^	2.19 ^d^	175.40 ^c^
***p* Value**							
Strain (S)		0.450	0.093	0.233	>0.001	>0.001	0.042
Heat stress (HS)		0.471	0.115	0.697	>0.001	0.031	>0.001
Boldo (B)		0.215	0.214	0.082	0.015	0.050	0.021
S × HS		0.576	0.147	0.240	0.034	0.014	0.040
S × B		0.391	0.258	0.331	0.012	>0.001	>0.001
HS × B		0.267	0.480	0.265	0.050	>0.001	0.051
S × HS × B		0.160	0.921	0.163	>0.001	0.043	>0.001
SEM		0.013	0.001	0.001	0.001	0.028	0.047
C.V.%		3.25	3.21	1.84	4.10	7.31	2.53

TN: thermoneutral temperature; HS: heat stress; B/C: benefit/cost; SEM: standard error of mean; C.V.%: coefficient of variation. Data presented as means and means in the same column with no superscript letters after them or with a common superscript letter following them are not significantly different (*p* < 0.05).
